# Body ownership gates tactile awareness by reshaping the somatosensory functional connectivity

**DOI:** 10.1073/pnas.2513533122

**Published:** 2025-12-18

**Authors:** Alberto Pisoni, Carlotta Fossataro, Alice Rossi Sebastiano, Marcella Romeo, Eleonora Arrigoni, Leonor Josefina Romero Lauro, Nadia Bolognini, Francesca Garbarini

**Affiliations:** ^a^Psychology Department and NeuroMi, University of Milano Bicocca, Milano 20126, Italy; ^b^MANIBUS Lab, Psychology Department, University of Turin, Turin 10124, Italy; ^c^MoMiLab, IMT School for Advanced Studies, Lucca 55100, Italy; ^d^Laboratory of Neuropsychology, IRCCS Istituto Auxologico Italiano, Milano 20122, Italy

**Keywords:** tactile awareness, body ownership, alpha-band connectivity, TMS-EEG

## Abstract

We all likely agree that tactile experience contributes to the emergence of the feeling of ownership over one’s own body. Is the opposite true? We answered this question by testing whether and how the sense of body ownership gates our tactile experience. In two experiments, we exploited a well-known multisensory illusion (Rubber Hand Illusion) to induce participants to feel a fake hand as belonging to their body, while their own hand was left in a disembodiment state (illusory-phases). After each illusory phase, a tactile stimulus was delivered to either the fake (embodied) hand or the real (disembodied) hand (testing-phases). Experiment 1 shows that the illusory phase significantly modulates the subjective feeling of touch experienced in the testing-phase, increasing tactile sensations when participants observed the fake (embodied) hand being touched (visual-touch), and decreasing them when the real (disembodied) hand was touched (real-touch). Experiment 2 investigated, by using TMS-EEG, the neural mechanism supporting this diametrical modulation of subjective feeling of touch, focusing on alpha-band oscillatory networks as the neural correlate of somatosensory awareness. S1 alpha-band connectivity fully matches the behavioral results, significantly increasing in visual-touch and decreasing in real-touch. In both experiments, a greater embodiment experienced in the illusory-phase significantly predicted higher behavioral and neurofunctional responses to visual-touch and lower responses to real-touch in the testing-phase. Altogether, our findings demonstrate that the sense of body ownership exerts a top-down modulation on tactile awareness and may do so by increasing or decreasing the strength of the somatosensory network involved in tactile awareness.

The sense of touch is strongly related to the bodily self, forming the boundary between one’s own and others’ bodies. Previous studies investigated the relationship between the sense of touch and body ownership [i.e., the feeling that body parts belong to us (1)] by addressing how somatosensory experiences arising from the skin contribute to discriminate one’s own body from the external world. This line of research corroborates the idea that tactile awareness (i.e., the conscious experience of tactile events) contributes to the emergence of the sense of body ownership, so that “I believe this body to be mine because I perceive tactile sensations on it.”^*^ Still unanswered remains the opposite question: “Do I feel tactile sensations on this body because I believe it to be mine?”.^*^ To answer this question, we capitalized on the well-known Rubber Hand Illusion [RHI (1)] able to modulate body ownership so that participants temporarily experience a fake hand as part of their own body (embodiment), while the own hand is subjected to a certain degree of disembodiment (2).

## Results and Discussion

**Fig. 1.**
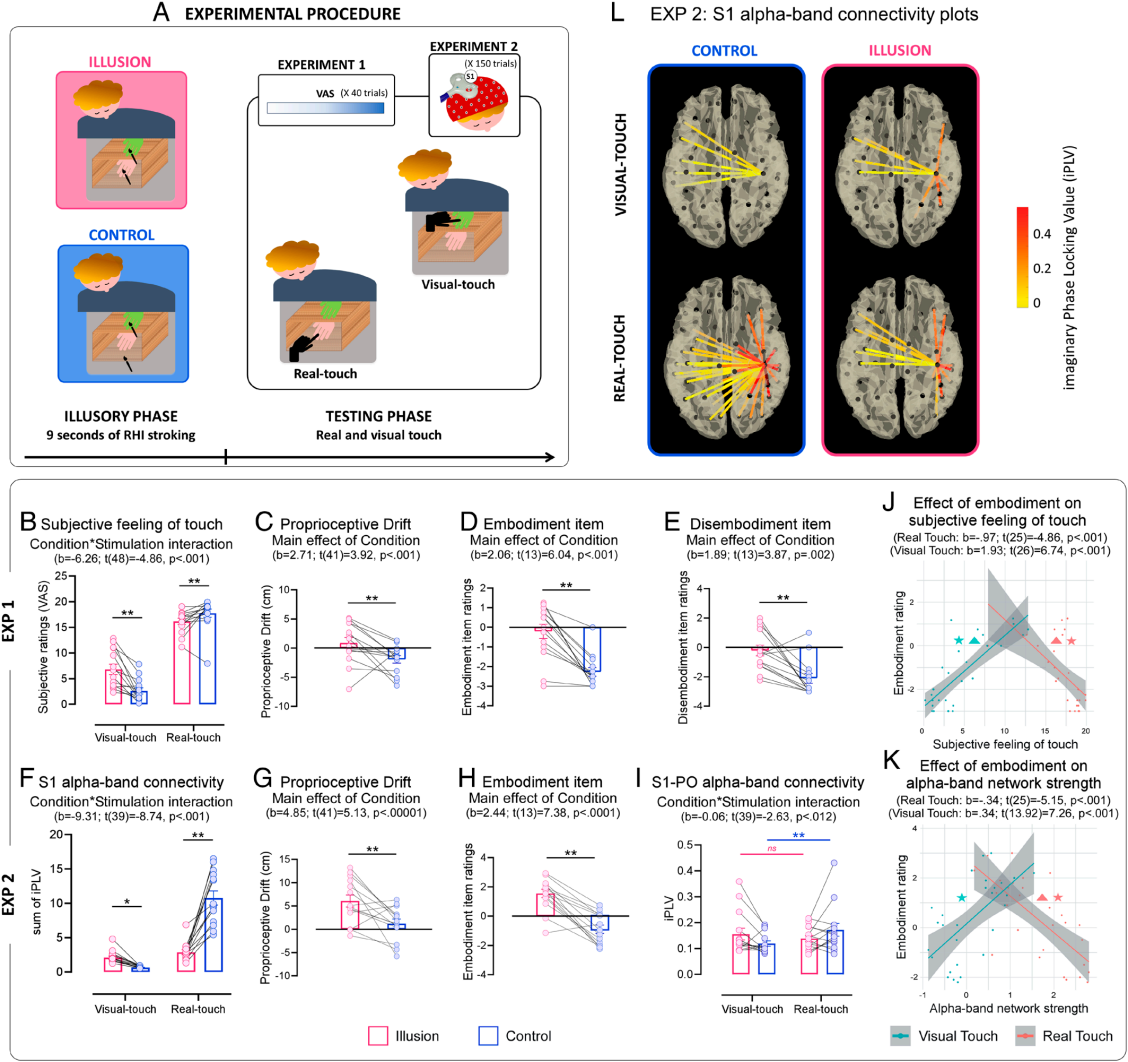
(*A*) Illusory-phase: RHI induction in Illusion (Synchronous) and Control (Asynchronous) conditions. Testing-phase: after visual-touch or real-touch, in Experiment 1, participants rated tactile sensations on a 0 to 20 VAS; in Experiment 2, EEG was recorded while TMS was delivered over S1. (*B*–*E*) results for Experiment 1. (*F*–*I*) results for Experiment 2. Error bars = SEM; dots = individual participants; iPLV = imaginary Phase Locking Values; *P < 0.05, **P < 0.001. (*J* and *K*) effect of the Embodiment on both subjective feeling of touch and alpha-band network strength. ★*P* < 0.001 after correcting for suggestibility, all models survived. ▲*P* < 0.05 after correcting for condition (synchronous/asynchronous), all models survived except the visual-touch model in Experiment 2. (*L*) Source-level iPLV between S1 and the other brain parcels in the 400 ms post-TMS windows.

In Experiment 1, we addressed whether an altered body ownership modulates subjective feelings of touch. Following repeated periods of illusion induction (illusory-phases), wherein the real and the fake hands were stroked either synchronously (illusion block) or asynchronously (control block), isolated touches were administered through a robotic hand on either the fake hand (visual-touch) or the real hand (real-touch), while participants had to rate their tactile feeling through a VAS (testing-phases). The RHI strength was monitored by embodiment/disembodiment items and proprioceptive drift (Fig. 1*A* and *SI Appendix*). A significant interaction between condition (illusion/control) and stimulation (real-/visual-touch) nicely indicates that, after the illusory-phases as compared to the control ones, tactile feelings experienced in the testing-phase were significantly modulated, with higher tactile ratings in visual-touch and lower tactile ratings in real-touch (Fig. 1*B*). Hence, the illusory feeling of body ownership, verified in our sample through standard illusion vs control comparisons (Fig. 1 *C*–*E*), gated the feeling of touch, fostering sensations of tactile events occurring on the embodied (fake) hand to the detriment of those occurring on the disembodied (real) hand. This resembles a fascinating phenomenon, occurring independently from the multisensory illusion, in brain-damaged patients with body awareness disorders. Indeed, patients presenting with *Pathological Embodiment* who misattribute another person’s hand as their own hand, even come to feel to be touched at the sight of someone else’s embodied hand being touched, along with showing enhanced skin conductance when stimuli are delivered to it (3). Conversely, patients with *Somatoparaphrenia*, who misattribute their own hand to someone else, show reduced arousal responses when stimuli are delivered to their own disembodied hand (4). Our paradigm replicates these pathological behaviors in healthy subjects, supporting the view that *we feel tactile sensations on a given body because we believe it to be ours*.

Experiment 2 explored the neurophysiological mechanisms of this body ownership-dependent modulation of tactile awareness. To this aim, we leveraged a TMS-EEG protocol to explore the functional connectivity between S1 and the rest of the brain (*SI Appendix*). We focused on S1 alpha-band connectivity being previously identified as the neural marker of somatosensory awareness as it has been found only when tactile stimulation reaches consciousness (5). By using a similar TMS-EEG protocol, Pisoni et al. (6) found a greater S1 alpha-band connectivity during real-touches on participants’ body as compared to visual-touches on someone else’ body. This result is in line with the role of S1 alpha-band connectivity in shaping somatosensory awareness (5), since the mere observation of a touch does not elicit tactile feelings in the observer. Here, we asked what happens to S1 alpha-band connectivity when, as in Experiment 1, the illusion leads the observer to feel tactile sensations following visual-touch, to the detriment of tactile sensations reported after real-touch. After each illusory-phases, EEG was recorded in the testing-phases while a TMS pulse was delivered over the right S1 50 ms after either a visual-touch occurring onto a fake hand, or a real-touch occurring onto the participants’ hand (in both illusion and control conditions; Fig. 1*A*). Network strength of S1 alpha-band connectivity showed a significant interaction between condition (illusion/control) and stimulation (real/visual) (Fig. 1*F*). While in the control condition, we observed greater alpha-band connectivity during real- than visual-touch (6), in the illusion condition such difference was abolished, as if the system no longer differentiated the neurophysiological responses between touches occurring onto the real or the fake (embodied) hand. Mirroring the behavioral results, illusory feeling of body ownership, verified through proprioceptive drift and embodiment item (Fig. 1 *G* and *H*), gated S1 alpha-band connectivity, which was significantly increased during visual-touch and significantly decreased during real-touch (Fig. 1*F*). In visual-touch trials, we found a strong increase in the S1 connectivity with an ipsilateral fronto-temporo-occipital network (Fig. 1*L*, *Top*), thought to facilitate the integration of visual information (from occipito-temporal areas) with higher-level cognitive processes (in the frontal lobe). Its stronger connectivity with S1 likely allows somatosensory processing of the visual touch, thus promoting tactile sensations starting from purely visual stimuli, in the absence of any tactile stimulation, [unlike previous studies always including tactile stimuli; e.g., (7)]. In real-touch trials, the reduced connectivity between S1 and an ipsilateral sensorimotor network (encompassing precentral and postcentral regions; Fig. 1*L*, *Bottom*) advocates a downregulation of motor (2) and somatosensory (8) systems following the illusion. Importantly, among those areas whose connectivity with S1 is significantly modulated, the contralateral parietal operculum, a crucial hub for tactile awareness (9), shows a diametrical modulation in visual- and real-touch (Fig. 1*I*). An additional analysis focusing on this connection showed that the stronger connectivity observed for real- compared to visual-touch in the control condition was abolished under the illusion condition, due to increased connectivity in visual-touch and decreased in real-touch, thus fostering tactile feeling by sight to the detriment of real tactile processing.

In both experiments, we directly tested whether individual differences in ownership strength (i.e., embodiment; disembodiment; drift) predicted the perceptual rating (Experiment 1) and the S1 alpha-band connectivity (Experiment 2) following visual- and real-touch, also controlling for suggestibility traits (*SI Appendix*). For the embodiment measure, correlational results nicely support the observed diametrical pattern, with greater embodiment values significantly predicting higher behavioral and neurofunctional responses to visual-touch and lower responses to real-touch (Fig. 1 *J* and *K*). As a possible limitation, we acknowledge that here, as in all other studies that exploit the RHI to manipulate the body ownership in healthy participants, illusion (synchronous) and control (asynchronous) conditions may differ in more than just ownership. They also involve differences in multisensory congruency, attention, and expectation, which could influence tactile awareness. Overall, our results endorse the diametrical tuning of the S1 alpha-band connectivity as the neural mechanism allowing the sense of body ownership to exert a top–down modulation on tactile awareness. A deeper understanding of this mechanism could be exploited to improve neuroprosthetic devices, enhancing patients’ compliance and proficiency in their use. Alongside this possible technological application, our data also contribute to the theoretical debate on self-consciousness, supporting a lower-order model of the self (10), in which it forms the foundation of tactile awareness.

## Materials and Methods

Ethics Committee of the University of Turin (N.510228) and Milano-Bicocca (N.445) approved the study. For complete details, see *SI Appendix*. Two volunteer samples, after expressing written consent, participated in Experiment 1 (n = 14) and Experiment 2 (n = 14). TMS-EEG was recorded with a 60-channel Nextim system. Data were analyzed by linear mixed effects models and are available at OSF https://osf.io/9brkc/?view_only=9f80dad3f0a1492197da24849fcaafa8 (11).

## Supplementary Material

Appendix 01 (PDF)

## Data Availability

Behavioral and neurophysiological data have been deposited in OSF (https://osf.io/9brkc/?view_only=9f80dad3f0a1492197da24849fcaafa8) (11).

## Footnotes

^*^The authors would like to express their gratitude to Frederique de Vignemont for her contribution in this conceptualization during the writing of the book “The Subject’s Matter. Self Consciousness and the Body.” de Vignemont, F., & Alsmith, A. (2017). *Representation and Mind*, 402.
