# Supplementary material for: Body ownership gates tactile awareness by reshaping the somatosensory functional connectivity
Source: Proc Natl Acad Sci U S A. 2025 Dec 18;122(51):e2513533122. doi: 10.1073/pnas.2513533122 (PMC12745685; doi:10.1073/pnas.2513533122)
Supplement: Supplementary file 1 — Appendix 01 (PDF) [file pnas.2513533122.sapp.pdf]

Supporting Information for

Body ownership gates tactile awareness by reshaping the somatosensory functional connectivity

Alberto Pisoni*, Carlotta Fossataro*, Alice Rossi Sebastiano, Marcella Romeo, Eleonora Arrigoni, Leonor Josefina Romero Lauro, Nadia Bolognini, Francesca Garbarini

Francesca Garbarini.
E-mail: francesca.garbarini@unito.it

This PDF file includes:

Supporting text

Supporting Information Text

PARTICIPANTS

Eleven healthy, right-handed volunteers [3 males, mean±(Standard Deviation, SD) age= 24.8±1.4 years] participated in a pilot experiment. This pilot experiment was conducted to test whether the ad-hoc modified version of the RHI protocol employed in both Experiment 1 and Experiment 2 was effective in inducing and maintaining the illusory feeling of ownership over the rubber hand even if visual- and real-touches were delivered in between the RHI stroking periods. Two different samples of fourteen healthy, right-handed volunteers took part in Experiment 1 (7 males, mean±SD age= 26.14±1.03 years) and in Experiment 2 (4 males, mean±SD age= 27±4.1 years). The sample size of both experiments was chosen based on the pilot experiment's results (see a-priori data analysis section below). Each participant completed an Adult Safety Screening Questionnaire(1) to assess potential contraindication to TMS, such as neurological, psychiatric, or other relevant medical problems(2) when TMS was applied. The study was performed in the behavioral lab of the University of Turin and in the TMS-EEG laboratory of the University of Milano-Bicocca, following the ethical standards of the Declaration of Helsinki. All participants were naïve to the purpose of the study.

METHODS DETAIL

Rubber Hand Illusion protocol. To alter the participants' sense of body ownership and address its role in shaping the subjective feeling of touch we exploited the well known RHI procedure. To this aim, we created an ad-hoc RHI protocol that was previously tested in the pilot experiment (see Pilot Experiment section below) and then adopted for both Experiment 1 and 2. Specifically, our RHI procedure is based on the classical design, in which synchronous stroking typically induces both embodiment of the rubber hand and disembodiment of the real hand. However, a key and novel feature of our study lies in the combination of this classic illusion induction (illusory-phase) with a tactile task (testing-phase), administered immediately after each illusion induction period (lasting 9 seconds) and repeated across multiple trials. Importantly, the tactile stimuli used for the tactile task were completely distinct from the stroking used to induce the illusion. In the illusion condition, the RHI was evoked by synchronously stroking the participants' index finger and the fake index finger from knuckle to fingertip, at an approximate frequency of 1 Hz(3). Asynchronous stroking of the participants' hand and the rubber hand was utilized as a control condition, in which strokes were delivered spatially and temporally out of phase between the two hands. During the RHI procedure, participants were seated in front of a box (7.5×14×10.5 cm) divided into two compartments. The rubber hand was placed on the upper shelf of the box, while the participants' left hand was placed on the lower shelf of the box to conceal it. The rubber hand and the participants' hand were vertically aligned (7.5 cm apart) and congruently positioned with respect to the participants' shoulder. A cloth covered the space between participants' shoulder and the fake arm to create the impression that the fake hand was attached to the participants' shoulder. To study the effect of the RHI procedure on tactile awareness (testing-phase), a tactile task was implemented in the RHI setting by employing two robotic hands providing tactile stimuli on either the participants' or fake hand. Indeed, besides both the participants' hand and the rubber hand, there were two human-like hands provided with servomotors allowing them to perform a flexion movement toward a target. This movement generated light touches on the dorsal surface of either the participants' or the rubber hand(4). These moving hands were custom-made and fully controlled by an Arduino script (http://www.arduino.cc) (see Figure 1, experimental procedure panel). Before starting, participants were familiarized with the setting and instructed on all procedures. As in the classical RHI procedure, participants were first blindfolded and asked to indicate the perceived position of their left index finger by pointing with their right index finger toward a vertical ruler attached to the right side of the box for 10 trials. The position marked on the ruler was averaged and referred to as proprioceptive judgments-pre. Then, participants were instructed to always maintain their gaze on the fake hand and remain still, while the RHI procedure was performed. In each trial, an experimenter stroked both the participants' and the rubber hand's index fingers for 9 seconds (either synchronously or asynchronously, according to the experimental block). When the stroking period expired, an acoustic signal prompted the experimenter to stop the stroking. After each stroking period three different experimental trials could occur: real-touch trial, wherein the robotic-hand beside the participants' hand touched it; visual-touch trial, wherein the robotic-hand beside the rubber hand touched it; catch trial, wherein no touch occurred and RHI measures (either implicit or explicit according to the experiment, see details below) were collected for monitoring the effectiveness of the illusion across trials. Note that, we shortened the illusion induction to 9 seconds, in order to collect the required number of trials for each experimental condition, enabling us to collect ratings of participants subjective feeling of touch (Experiment 1), and S1 alpha-band connectivity (Experiment 2). Importantly, our ad-hoc RHI design allowed us to study the role of body ownership in shaping real-touch processing but also purely visual-touch processing in the absence of any actual tactile stimulation, differing our approach from those adopted in previous studies(5–8). At the end of each experimental block (synchronous or asynchronous), participants expressed 10 proprioceptive judgments again, and their average was referred to as proprioceptive judgements-post. Illusion (synchronous) and control (asynchronous) conditions were collected in separate blocks. Block order was counterbalanced between subjects, so that half of the participants performed the illusion condition and then the control one, and vice-versa for the other half.

Pilot Experiment. The goal of the Pilot Experiment was to verify whether the modified version of the RHI procedure adopted in both Experiment 1 and Experiment 2 was still effective in inducing and maintaining the illusion, even if visual- and real-touches were delivered in between the RHI stroking periods. A total of 24 trials per block was performed, alternating the RHI stroking of the participants' and the rubber hands according to the experimental condition (illusion and control) for 9 seconds, with 10

visual-touch trials, 10 real-touch trials and 4 catch trials. Each block lasts about 8-10 min. During catch trials, both implicit (i.e., proprioceptive judgment) and explicit (i.e., embodiment item) RHI measures were collected. The implicit one consisted of an eye-closed pointing ballistic movement of the right hand towards the aforementioned vertical ruler, signaling the estimated position of one's own hand (the average was referred to as proprioceptive judgments-catch). The explicit measure consisted in verbally rating the agreement with an embodiment item (i.e., "I felt as if the rubber hand was my hand") on a Likert scale ranging from -3 to +3 (adapted from(9, 10)). Trials order was randomized and controlled by an E-Prime script (E-prime software 2.0, Psychology Software Tools, Inc, www.psychotoolbox.org).

Experiment 1. Experiment 1 was conducted to test whether the illusory feeling of ownership of the rubber hand affected the participants' subjective feeling of touch. The RHI procedure was performed as described above. Crucially, when the robotic hand touched the participants' (real-touch, 20 trials) or the rubber hand (visual-touch, 20 trials), participants were asked to rate their feeling of having been touched (i.e., subjective rating of touch) on a visual analogue scale (VAS)(11, 12) in which 0 means "no feeling at all" and 20 means "clear feeling of touch". During catch trials (4 trials) both implicit and explicit RHI measures were collected. The implicit one consisted of an eye-closed pointing ballistic movement of the right hand towards the aforementioned vertical ruler, signaling the estimated position of one's own hand (the average was referred to as proprioceptive judgments-catch). The explicit measure consisted in verbally rating the agreement with an embodiment item (i.e., "I felt as if the rubber hand was my hand") and a disembodiment item (i.e., "I felt as if my hand had disappeared") on a Likert scale ranging from -3 to +3 [adapted from(9, 10)]. Trials order was randomized and controlled by an E-Prime script (E-prime software 2.0, Psychology Software Tools, Inc, www.psychotoolbox.org).

Experiment 2. Experiment 2 was conducted to measure the neural markers of tactile awareness modulation following the RHI. TMS-EEG was applied during the very same ad-hoc RHI protocol described above, with two RHI stroking blocks (illusion and control conditions) collected separately. In each block, a total of 160 trials were collected, and each trial consisted of a RHI stroking period lasting 9 s immediately followed either by real-touch trials (75), visual-touch trials (75), or catch-trials (10). When the robotic hand touched the participants' or the rubber hand, a signal was prompted through a touch controlling device (Makey Makey, https://makeymakey.com/), triggering a TMS pulse over the participants' right S1 hand area 50 ms after the tactile stimulus delivery (Figure 1, experimental procedure panel). This SOA was chosen according to previous TMS-EEG evidence highlighting a different S1 response at this latency following perceived and observed tactile events, in terms of EEG functional connectivity(13). In Catch trials, the TMS pulse was not delivered, and RHI measures were collected to monitor the effectiveness of the illusion. Specifically, a proprioceptive judgement and the agreement with the embodiment item were provided by the participants. Note that, because of time constraints, we selected only a single embodiment item as an explicit measure. The inter-trial interval was jittering between 100-500 ms for a total duration of 12.6-13s for each trial (catch trials 19.6-20 s; see Figure 1, experimental procedure panel). Each block lasted approximately 35 min, for a total experimental time of 70 min.

TMS-EEG based functional connectivity. The integration of TMS and EEG techniques allows for a direct probe of the cortical connectivity of brain regions (14, 15). Although EEG provides excellent temporal resolution, it is limited in its capacity to infer causality or precisely identify the cortical sources of recorded activity. TMS overcomes these limitations by delivering controlled perturbations to targeted cortical regions, enabling researchers to establish direct relationships between neural activity and concurrent cognitive or behavioral processes. In turn, EEG extends the capabilities of TMS by capturing the brain's immediate electrophysiological responses, both at the stimulation site and in remote, functionally connected regions across the cortex(14). Together, TMS and EEG allow for direct, real-time mapping of how neural activity propagates through cortical networks, providing both spatial and temporal precision. This integration offers a powerful framework for investigating the dynamics of inter-regional communication, revealing when and how activation in one area influences activity in others. Furthermore, functional connectivity analysis of TMS-EEG data can uncover qualitative aspects of the triggered activity across cortical regions. TMS actively initiates oscillatory dynamics, whose propagation to distant areas reflects the communication status and state dependence of the targeted functional network. The distribution and coherence of these evoked oscillations depend both on the intrinsic properties of the stimulated region and on its functional embedding within the broader network, providing a powerful means of probing the mechanisms of interregional communication, both at rest and during task performance(16).

TMS stimulation. TMS was delivered using an Eximia™ TMS stimulator (Nexstim™, Helsinki, Finland) with a biphasic focal figure-of-eight 70-mm coil. As stimulation target, the right S1 hand area was chosen, following the MNI coordinates 47, -32, 59 (17) as in previous TMS and TMS-EEG studies targeting this region (Pisoni et al., 2018; Bolognini et al., 2014, 2011, 2010a; Rocchi et al., 2016). These coordinates were localized on normalized individual MRIs: after the acquisition of a high-resolution (1x1x1 mm) structural magnetic resonance image (MRI) using a 3T Intera Philips body scanner (Philips Medical Systems, Best, NL), the images were normalized in SPM 12 (https://www.fil.ion.ucl.ac.uk/spm/software/spm12/), and the MNI coordinates were localized on these images. This information was then used to perform the neuronavigation on the individual MRIs through an integrated Navigated Brain Stimulation (NBS) system (Nexstim™, Helsinki, Finland). This system uses an infrared-based frameless stereotaxy technique to map the subject's head and coil position within the reference space of the individual's MRI. With this NBS system it was also possible to monitor online, during the stimulation, the coil's position, orientation and inclination, ensuring the reproducibility of the stimulation protocol across sessions. Furthermore, the NBS system estimated the intensity (V/m) of the intracranial electric field induced by TMS at the stimulation hotspot, considering both individual head

and brain shapes, as well as the distance from the scalp and the coil's position. To determine stimulation intensity, we adopted a functional procedure(18), setting the stimulator output according to the recorded TMS evoked response. Specifically, we chose an intensity that elicited a cortical response of at least 6µV. The mean estimated electric field at the stimulation target was 98.6 V/m (SD ± 10.6), corresponding to a mean stimulation intensity expressed as a percentage of the maximal stimulator output (MSO) of 59.6 (SD ± 2.6). Within each subject, the MSO intensity was kept fixed across TMS-EEG recordings. The coil was placed tangentially to the scalp, and no MEPs of the contralateral upper limbs were reported for any subject.

EEG data recording. For each participant, two TMS-EEG recordings of 160 pulses each were performed. EEG signal was continuously acquired using a TMS compatible 60-channels amplifier (Nexstim Ltd., Helsinki, Finland). A proprietary sample-and-hold circuit held the amplifier output constant from 100s pre to 2ms post-TMS pulse(19). The ground and reference electrodes were placed over the subject's forehead, while the electrooculogram (EOG) was recorded by two electrodes placed near the eyes. TMS noise was masked by continuously playing an audio track created by shuffling the TMS discharge noise(20–23), preventing the emergence of auditory evoked potentials linked to the coil click(24). Electrodes' impedance was kept below 5 kΩ, and EEG sampling rate was 1450 Hz. Data pre-processing was performed using Matlab R2012a (Mathworks, Natick, MA, USA). First, a band-pass filter between 1 and 45 Hz was applied. Second, EEG signal was split into epochs 800 ms before and 800 ms after the TMS pulse, down-sampling EEG signals to 725 Hz. Third, artifact rejection was performed by removing trials with excessive artifacts by visual inspection(25), and TMS Evoked Potentials (TEPs) were computed by averaging selected artifact-free single epochs. The spherical interpolation function in EEGLAB(26) was used to interpolate bad channels. TEPs were then average-referenced and baseline corrected between -800 ms and -650 ms before TMS pulse, and finally, to remove remaining muscular, ocular, or magnetic artifacts, an Independent Component Analysis (ICA) was applied. The average number of trials considered in the analysis was 68.8±4 for visual-touch control trials, 70.3±4 for visual-touch illusion trials, 68.5±5.5 for real-touch control trials, and 68.7±4.4 for real-touch illusion trials.

QUANTIFICATION AND STATISTICAL ANALYSIS

A-priori power analysis . G-power software (www.psycho.uniduesseldorf.de/abteilungen/aap/gpower3) was used to estimate, in an a priori analysis, the sample size in a paired t-test (two tailed), using the Cohens' d = 1.07; = 0.05; Power (1- err prob) = 0.98, based on the data collected in the pilot experiment. A total sample size of 14 subjects was indicated. Furthermore, this sample is in agreement with a previous study addressing the RHI-dependent modulation of the somatosensory system(27) and a TMS-EEG study investigating the electrophysiological correlates of observed-touch(13, 28, 29).

RHI measures analysis (Pilot Experiment, Experiment 1 and 2). In all experiments, the Proprioceptive Drift was calculated as the difference between the proprioceptive judgment collected before the RHI procedure (proprioceptive judgments-pre) and both the proprioceptive judgment collected after the RHI (proprioceptive judgments-post) and the proprioceptive judgment collected during the catch trials (proprioceptive judgments-catch). The obtained values were taken as a measure of proprioceptive relocation (i.e., proprioceptive shift toward the rubber hand) and analyzed with the statistical software R (version 4.2.0, R Development Core Team, 2022) through a series of Linear mixed effects models(30) with condition (2 levels: illusion; control) and time (2 levels: catch; post) as within subject factors, while the random effects structure included the by Subject intercept (LMER procedure in "lme4" R package, version 1.1.31,(31). A series of likelihood ratio tests was used to assess the inclusion of fixed effects that significantly increased the model's goodness of fit by means of a forward stepwise inclusion procedure (30). We reported the parameters of the final best fitting models with significance levels based on Satterthwaite's degrees of freedom approximation in "lmerTest" R package (version 3.1.3,(32)). Post hoc procedures were carried out on the best-fitting final model with the "phia" R package (version 0.2.1,(33)), applying Bonferroni-Holmes correction for multiple comparisons. The ratings to the embodiment (in Pilot, Experiment 1 and 2) and/or disembodiment (in Experiment 1) items were analyzed using the same procedures but including only the fixed factor of Condition in the models. Note that LMMs have been chosen since are optimal also for Likert-like measures (34, 35).

Pilot experiment. The Pilot Experiment results confirmed that the illusion persisted despite our modified RHI protocol, thus validating our experimental design. Regarding the Proprioceptive Drift, there was a significant main effect of RHI condition (b=3.44; t(30)= 3.55; p= .001), indicating a greater drift in synchronous (2.8cm) as compared to asynchronous (.77cm) condition, independently from the time of measurement. Indeed, time effect and its interaction with condition were not significant (b=-0.76; t(30)=-.78; p= 44 and b=-.35; t(30)=.26; p= .8 respectively). As concerning the embodiment item, the significant effect of condition (b=2.3; t(10)=4.77; p< .001) indicated a significantly greater illusory feeling of ownership over the fake hand in the illusion (0.29pts) as compared to the control condition (-2.02pts).

Experiment 1

Subjective ratings of touch. As for the RHI measure analysis, linear mixed effects models were used also for subjective ratings of touch as the main statistical procedure(36), employing the statistical programming environment R and the same aforementioned packages(37). As fixed effects, stimulation (factorial, 2 levels: visual-touch vs real-touch), condition (factorial, 2 levels: illusion vs control), and their interactions were tested. Concerning the random effect structure, a by-subject random intercept was included. We report the parameters of the final best-fitting models with significance levels based on Satterthwaite's degrees of freedom approximation. Moreover, to directly contrast single levels of the significant interactions and main effects, post hoc procedures were carried out on the best fitting final model applying Bonferroni-Holmes correction for multiple comparisons.

Experiment 2

EEG data analysis. EEG data analysis was performed using the FieldTrip Matlab toolbox(38).

Alpha-band connectivity . Concerning the functional connectivity analysis, this was computed at the source level. The forward model was created starting from a Boundary Element Model (BEM) obtained by segmenting a subject MRI into five standard tissues (Gray and white matters, CSF, Skull and Scalp). The head model was then computed assigning standard conductivity values for the scalp, skull and brain compartments(39, 40). Source space was defined performing a cortical reconstruction and volumetric segmentation of the grey matter with the Freesurfer image analysis suite(41), down-sampled to 8193 cortical sources and re-aligned to the head model space. Finally, individual lead field matrices were computed by aligning this forward model with the individual electrodes' positions, which were recorded during each TMS-EEG session, to create a spatial filter matrix. This model was also used to compute the spatial filter matrix, i.e., the inverse of the lead field matrix. Source reconstruction of the EEG time-series was conducted with the LCMV method implemented in Fieldtrip toolbox(42–44) for solving the inverse modeling. Source signals were then segmented into 89 regions of interest (ROIs) according to the AAL brain atlas(45). This procedure fairly approximates spatial distribution of functional connectivity maps while keeping the False Positive Rate low (46). The computed source time-series were then averaged to index each ROI source activation(47). Functional connectivity was performed for the right S1 source space-time series. Specifically, imaginary Phase Locking Value [iPLV(48, 49)] was computed between S1 and the other brain parcels for (8-12Hz) band for each of the four experimental conditions, since this was the frequency of interest in discriminating somatosensory processing conditions and real- and visual-touch differences(13, 48). In detail, we chose to estimate S1-related alpha-band oscillatory network in line with a previous study(13) in which, using the same TMS-EEG approach, we documented a significant increase of S1 alpha-band connectivity only in the real touch condition. Accordingly, functional connectivity in this frequency band has been specifically related to conscious experience of tactile stimulation, i.e., when subjects are aware of its occurrence(48, 50), unlike other aspects of somatosensory processing. The same analysis was also performed on surrogate datasets created by shuffling the phase of the source reconstructed time series for each experimental condition. To reduce the risk that spurious connectivity could be included in our results, real data were compared with the surrogate computing a t-test performed on each connectivity pair (88, right S1 with the other 88 brain parcels), and corrected for multiple comparison based on 2000 permutation approach, implemented in Matlab, with a significance level of p= 0.05(13). Surviving connections were then plotted to highlight the resulting functional connectivity between the right S1 and the rest of the brain parcels (Figure 1C and Table S1). To compare the resulting global connectivity patterns across experimental conditions, the network strength was computed as the sum of the iPLV of the resulting connections. For the analysis we employed the statistical programming environment R (R Development Core Team, 2022) with the same aforementioned packages (37). Linear mixed effects models were used as the main statistical procedure(36). The dependent variable (i.e., network strength) were submitted to a series of linear mixed effects regression (37) and a series of likelihood ratio tests was used to assess the inclusion of fixed effects that significantly increased the model's goodness of fit by means of a forward stepwise inclusion procedure. As fixed effects, stimulation (factorial, 2 levels: visual-touch vs real-touch), condition (factorial, 2 levels: illusion vs control), and their interactions were tested. Concerning the random effect structure, a by-subject random intercept was included. We report the parameters of the final best fitting models with significance levels based on Satterthwaite's degrees of freedom approximation. Moreover, to directly contrast single levels of the significant interactions and main effects, post hoc procedures were carried out on the best fitting final model, applying Bonferroni-Holmes correction for multiple comparisons. The network strength analysis revealed a diametrical modulation of the S1 alfa-band connectivity with a variety of brain regions (see main text and Table S1) among which the Parietal Operculum (PO) stood out. Thus, given the role of PO in tactile awareness(51, 52), we specifically extracted the iPLV between S1 and PO for each experimental condition and performed a supplementary analysis on these values by employing the very same statistical approach of the network strength analysis.

Control Analyses

To directly test whether individual differences in body ownership strength during the illusory-phase predicted the tactile rating (Experiment 1) and the S1 alpha-band functional connectivity (Experiment 2) recorded in the testing-phase following visual- and real-touch , we run a series of linear mixed effects regressions on the dependent variables (i.e., subjective ratings of touch for Experiment 1, Network strength and S1-PO connections for Experiment 2) with a measure of illusory body ownership modulation (separately: drift, embodiment (Experiment 1 and 2) and disembodiment (Experiment 1) as fixed effects. Models were separately run for real and visual touches. For all these models we also further controlled for the condition by adding it as fixed factor (illusion vs. control). Only models with the embodiment ratings as a fixed effect showed significant results in both Experiment 1 and 2 and are presented in the main text (see Figure 1 F-M). Note that, in the Experiment 1, both visual- and real-touches models survived after controlling for condition. In the Experiment 2, only the real-touch model survived after controlling for condition. Finally, to control for individual suggestibility traits, known to influence susceptibility to RHI(53), we retrospectively administered the Short Suggestibility Scale [SSS,(54)] to participants from Experiment 1 (14/14) and Experiment 2 (12/14). The SSS is a short version form of the Multidimensional Iowa Suggestibility Scale(55), which is a self-report scale developed to assess the tendency to accept and internalize external influences. The scale consists of 21 items that are divided into five subscales: consumer suggestibility, persuadability, sensation contagion, physiological reactivity and conformism. To evaluate whether SSS, besides embodiment, could predict the observed effects on the collected dependent variables from both Experiment 1 and 2, we re-run the same models adding as covariate scores at the SSS. Note that, in both

Experiment 1 and Experiment 2, both visual- and real-touches models survived after controlling for SSS. Furthermore, this variable never predicted the dependent variable, as it never resulted significant in any model.

Labels corresponding to the brain parcels showing significant alpha-band connectivity with S1 surviving multiple comparison correction

Visual-Touch Control. "Frontal Inf Oper L"; "Frontal Inf Tri L"; "Rolandic Oper L"; "Thalamus R"; "Temporal Sup R"

Visual-Touch Illusion. "Frontal Inf Oper L"; "Frontal Inf Tri R"; "Rolandic Oper L"; "Calcarine R"; "Fusiform R"; "Postcentral L"; "Angular R"; "Heschl R"; "Temporal Pole Sup L"; "Temporal Pole Sup R"; "Temporal Pole Mid L"

Real-Touch Control. "Precentral L"; "Precentral R"; "Frontal Sup R"; "Frontal Mid R"; "Rolandic Oper L"; "Rolandic Oper R"; "Supp Motor Area R"; "Insula L"; "Insula R"; "Cingulum Mid L"; "Cingulum Mid R" ; "Cingulum Post L" ; "Cingulum Post R"; "Hippocampus L"; "ParaHippocampal L" ; "Amygdala L"; "Calcarine L"; "Calcarine R"; "Cuneus L"; "Cuneus R"; "Lingual L"; "Lingual R"; "Occipital Sup L"; "Occipital Sup R"; "Occipital Mid R"; "Fusiform L"; "Postcentral L"; "Parietal Sup L"; "Parietal Sup R"; "Parietal Inf L"; "Parietal Inf R"; "SupraMarginal L"; "SupraMarginal R"; "Angular L"; "Angular R"; "Precuneus L"; "Precuneus R"; "Paracentral Lobule L"; "Paracentral Lobule R"; "Putamen R"; "Pallidum L"; "Pallidum R"; "Thalamus L"; "Thalamus R"; "Heschl L"; "Temporal Sup R"; "Temporal Pole Sup L"; "Temporal Pole Sup R"; "Temporal Mid R"; "Temporal Inf L"

Real Touch Illusion. "Precentral L"; "Frontal Mid R"; "Frontal Inf Oper L"; "Frontal Inf Oper R"; "Frontal Inf Tri L"; "Frontal Inf Tri R"; "Rolandic Oper L"; "Occipital Inf R"; "Fusiform R"; "Postcentral L"; "SupraMarginal R"; "Angular R"; "Thalamus L"; "Heschl L"; "Temporal Pole Sup L"; "Temporal Pole Sup R"; "Temporal Pole Mid L"; "Temporal Inf L"

References

1. J. C. Keel, M. J. Smith, E. M. Wassermann, Letter to the Editor. A safety screening questionnaire for transcranial magnetic stimulation. Clin Neurophysiol 112, 720 (2000).

2. S. Rossi, et al., Safety and recommendations for TMS use in healthy subjects and patient populations, with updates on training, ethical and regulatory issues: Expert Guidelines. Clinical Neurophysiology [Preprint] (2021). Available at: https://doi.org/10.1016/j.clinph.2020.10.003 [Accessed 29 May 2023].

3. M. Galigani, C. Fossataro, P. Gindri, M. Conson, F. Garbarini, Monochannel Preference in Autism Spectrum Conditions Revealed by a Non-Visual Variant of Rubber Hand Illusion. J Autism Dev Disord (2021). https://doi.org/10.1007/s10803-021-05299-9.

4. C. Fossataro, et al., The sense of body-ownership gates cross-modal improvement of tactile extinction in brain-damaged patients. Cortex 127, 94–107 (2020).

5. S. Dietz, A. Nair, J. Medina, The influence of vision on tactile stroke perception: Evidence from a novel mirror box illusion. J Vis 25, 2239–2239 (2025).

6. K. Kilteni, H. H. Ehrsson, Body ownership determines the attenuation of self-generated tactile sensations. Proceedings of the National Academy of Sciences 114, 8426–8431 (2017).

7. R. Zopf, J. A. Harris, M. A. Williams, The influence of body-ownership cues on tactile sensitivity. Cogn Neurosci 2, 147–154 (2011).

8. F. Cardini, M. R. Longo, P. Haggard, Vision of the Body Modulates Somatosensory Intracortical Inhibition. Cerebral Cortex 21, 2014–2022 (2011).

9. M. Botvinick, J. D. Cohen, Rubber hands "feel" touch that eyes see. Nature 391, 756 (1998).

10. C. Fossataro, V. Bruno, S. Giurgola, N. Bolognini, F. Garbarini, Losing my hand. Body ownership attenuation after virtual lesion of the primary motor cortex. European Journal of Neuroscience 48, 2272–2287 (2018).

11. M. Fusaro, G. Tieri, S. M. Aglioti, Influence of cognitive stance and physical perspective on subjective and autonomic reactivity to observed pain and pleasure: An immersive virtual reality study. Conscious Cogn 67, 86–97 (2019).

12. V. Nicolardi, et al., Taking an embodied avatar's perspective modulates the temporal dynamics of vicarious pain and pleasure: a virtual reality and EEG study. Soc Cogn Affect Neurosci 20, 35 (2025).

13. A. Pisoni, L. J. Romero Lauro, A. Vergallito, O. Maddaluno, N. Bolognini, Cortical dynamics underpinning the self-other distinction of touch: A TMS-EEG study. Neuroimage 178, 475–484 (2018).

14. M. Massimini, Breakdown of Cortical Effective Connectivity During Sleep. Science (1979) 309, 2228–2232 (2005).

15. R. J. Ilmoniemi, et al., Neuronal responses to magnetic stimulation reveal cortical reactivity and connectivity. Neuroreport 8, 3537–3540 (1997).

16. E. Arrigoni, A. Pisoni, Analysis of brain connectivity through TMS-EEG: A review of current approaches and future directions. (2025). `https://doi.org/10.31234/OSF.IO/5CEMA_V1`.

17. M. Boakye, S. C. Huckins, N. M. Szeverenyi, B. I. Taskey, C. J. Hodge, Functional magnetic resonance imaging of somatosensory cortex activity produced by electrical stimulation of the median nerve or tactile stimulation of the index finger. J Neurosurg 93, 774–783 (2000).

18. U. Ziemann, et al., TMS and drugs revisited 2014. Clinical Neurophysiology 126, 1847–1868 (2015).

19. J. Virtanen, J. Ruohonen, R. Näätänen, R. J. Ilmoniemi, Instrumentation for the measurement of electric brain responses to transcranial magnetic stimulation. Med Biol Eng Comput 37, 322–326 (1999).

20. S. Casarotto, et al., EEG responses to TMS are sensitive to changes in the perturbation parameters and repeatable over time. PLoS One 5 (2010).

21. L. J. Romero Lauro, et al., TDCS increases cortical excitability: Direct evidence from TMS-EEG. Cortex 58, 99–111 (2014).

22. M. Massimini, Breakdown of Cortical Effective Connectivity During Sleep. Science (1979) 309, 2228–2232 (2005).

23. A. Pisoni, et al., Cognitive Enhancement Induced by Anodal tDCS Drives Circuit-Specific Cortical Plasticity. Cerebral Cortex 1–9 (2017). https://doi.org/10.1093/cercor/bhx021.

24. E. M. ter Braack, C. C. de Vos, M. J. A. M. van Putten, Masking the Auditory Evoked Potential in TMS-EEG: A Comparison of Various Methods. Brain Topogr 28, 520–528 (2015).

25. A. G. Casali, S. Casarotto, M. Rosanova, M. Mariotti, M. Massimini, General indices to characterize the electrical response of the cerebral cortex to TMS. Neuroimage 49, 1459–1468 (2010).

26. A. Delorme, S. Makeig, EEGLAB: An open source toolbox for analysis of single-trial EEG dynamics including independent component analysis. J Neurosci Methods 134, 9–21 (2004).

27. D. Zeller, V. Litvak, K. J. Friston, J. Classen, Sensory Processing and the Rubber Hand Illusion-An Evoked Potentials Study. J Cogn Neurosci 27, 573–582 (2015).

28. G. Guidali, E. Arrigoni, N. Bolognini, A. Pisoni, M1 large-scale network dynamics support human motor resonance and its plastic reshaping. Neuroimage 308, 121082 (2025).

29. V. Bianco, E. Arrigoni, F. Di Russo, L. J. Romero Lauro, A. Pisoni, Top-down reconfiguration of SMA cortical connectivity during action preparation. iScience 26, 107430 (2023).

30. R. H. Baayen, D. J. Davidson, D. M. Bates, Mixed-effects modeling with crossed random effects for subjects and items. J Mem Lang 59, 390–412 (2008).

31. D. Bates, M. Mächler, B. M. Bolker, S. C. Walker, Fitting Linear Mixed-Effects Models Using lme4. J Stat Softw 67, 1–48 (2015).

32. A. Kuznetsova, P. B. Brockhoff, R. H. B. Christensen, lmerTest Package: Tests in Linear Mixed Effects Models. J Stat Softw 82, 1–26 (2017).

33. H. De Rosario-Martinez, CRAN: Package phia. (2015). Available at: https://cran.r-project.org/web/packages/phia/index.html [Accessed 10 August 2025].

34. G. Norman, Likert scales, levels of measurement and the "laws" of statistics. Advances in Health Sciences Education 15, 625–632 (2010).

35. E. Gibson, S. Piantadosi, K. Fedorenko, Using mechanical turk to obtain and analyze English acceptability judgments. Linguistics and Language Compass 5, 509–524 (2011).

36. R. H. Baayen, D. J. Davidson, D. M. Bates, Memory and Language Mixed-effects modeling with crossed random effects for subjects and items. J Mem Lang 59, 390–412 (2008).

37. D. M. Bates, Parsimonious Mixed Models. (2015).

38. R. Oostenveld, P. Fries, E. Maris, J. M. Schoffelen, FieldTrip: Open source software for advanced analysis of MEG, EEG, and invasive electrophysiological data. Comput Intell Neurosci 2011 (2011).

39. M. Fuchs, J. Kastner, M. Wagner, S. Hawes, J. S. Ebersole, A standardized boundary element method volume conductor model. Clinical Neurophysiology 113, 702–712 (2002).

40. J. Vorwerk, et al., A guideline for head volume conductor modeling in EEG and MEG. Neuroimage 100, 590–607 (2014).

41. B. Fischl, FreeSurfer. Neuroimage [Preprint] (2012).

42. B. D. Van Veen, W. Van Drongelen, M. Yuchtman, A. Suzuki, Localization of brain electrical activity via linearly constrained minimum variance spatial filtering. IEEE Trans Biomed Eng 44, 867–880 (1997).

43. K. Mahjoory, et al., Consistency of EEG source localization and connectivity estimates. Neuroimage 152, 590–601 (2017).

44. T. Popov, R. Oostenveld, J. M. Schoffelen, FieldTrip made easy: An analysis protocol for group analysis of the auditory steady state brain response in time, frequency, and space. Front Neurosci 12 (2018).

45. N. Tzourio-Mazoyer, et al., Automated anatomical labeling of activations in SPM using a macroscopic anatomical parcellation of the MNI MRI single-subject brain. Neuroimage 15, 273–89 (2002).

46. D. Brkić, et al., The impact of ROI extraction method for MEG connectivity estimation: Practical recommendations for the study of resting state data. Neuroimage 284, 120424 (2023).

47. A. Hillebrand, G. R. Barnes, J. L. Bosboom, H. W. Berendse, C. J. Stam, Frequency-dependent functional connectivity within resting-state networks: An atlas-based MEG beamformer solution. Neuroimage 59, 3909 (2012).

48. J. Hirvonen, S. Palva, Cortical localization of phase and amplitude dynamics predicting access to somatosensory awareness. Hum Brain Mapp 37, 311 (2016).

49. J. P. Lachaux, E. Rodriguez, J. Martinerie, F. J. Varela, Measuring phase synchrony in brain signals. Hum Brain Mapp 8, 194–208 (1999).

50. S. Palva, K. Linkenkaer-Hansen, R. Näätänen, J. M. Palva, Early neural correlates of conscious somatosensory perception. Journal of Neuroscience 25, 5248–5258 (2005).

51. P. Avanzini, et al., Four-dimensional maps of the human somatosensory system. Proceedings of the National Academy of Sciences 113, E1936–E1943 (2016).
52. M. Del Vecchio, et al., Tonic somatosensory responses and deficits of tactile awareness converge in the parietal operculum. Brain 144, 3779–3787 (2021).
53. P. Lush, et al., Trait phenomenological control predicts experience of mirror synaesthesia and the rubber hand illusion. Nat Commun 11, 1–10 (2020).
54. M. T. Liuzza, et al., Italian adaptation of the Multidimensional Iowa Suggestibility Scale (MISS). PeerJ 12, e17145 (2024).
55. R. Kotov, S. Bellman, D. Watson, Multidimensional Iowa Suggestibility Scale (MISS) , Stony Brook University, Ed. (2004).
